# Minor Abnormalities of Testis Development in Mice Lacking the Gene Encoding the MAPK Signalling Component, MAP3K1

**DOI:** 10.1371/journal.pone.0019572

**Published:** 2011-05-03

**Authors:** Nick Warr, Debora Bogani, Pam Siggers, Rachel Brixey, Hilda Tateossian, Asha Dopplapudi, Sara Wells, Michael Cheeseman, Ying Xia, Harry Ostrer, Andy Greenfield

**Affiliations:** 1 Mammalian Genetics Unit, Medical Research Council, Harwell, Oxfordshire, United Kingdom; 2 The Mary Lyon Centre, Medical Research Council, Harwell, Oxfordshire, United Kingdom; 3 Department of Environmental Health and Center of Environmental Genetics, School of Medicine, University of Cincinnati, Cincinnati, Ohio, United States of America; 4 Human Genetics Program, New York University School of Medicine, New York, New York, United States of America; National University of Singapore, Singapore

## Abstract

In mammals, the Y chromosome is a dominant male determinant, causing the bipotential gonad to develop as a testis. Recently, cases of familial and spontaneous 46,XY disorders of sex development (DSD) have been attributed to mutations in the human gene encoding mitogen-activated protein kinase kinase kinase 1, MAP3K1, a component of the mitogen-activated protein kinase (MAPK) signal transduction pathway. In individuals harbouring heterozygous mutations in *MAP3K1,* dysregulation of MAPK signalling was observed in lymphoblastoid cell lines, suggesting a causal role for these mutations in disrupting XY sexual development. Mice lacking the cognate gene, *Map3k1*, are viable and exhibit the eyes open at birth (EOB) phenotype on a mixed genetic background, but on the C57BL/6J genetic background most mice die at around 14.5 dpc due to a failure of erythropoiesis in the fetal liver. However, no systematic examination of sexual development in *Map3k1*-deficient mice has been described, an omission that is especially relevant in the case of C57BL/6J, a genetic background that is sensitized to disruptions to testis determination. Here, we report that on a mixed genetic background mice lacking *Map3k1* are fertile and exhibit no overt abnormalities of testis development. On C57BL/6J, significant non-viability is observed with very few animals surviving to adulthood. However, an examination of development in *Map3k1*-deficient XY embryos on this genetic background revealed no significant defects in testis determination, although minor abnormalities were observed, including an increase in gonadal length. Based on these observations, we conclude that MAP3K1 is not required for mouse testis determination. We discuss the significance of these data for the functional interpretation of sex-reversing *MAP3K1* mutations in humans.

## Introduction

Disorders of sex development (DSD) comprise a large number of cases in which development of chromosomal, gonadal or anatomical sex is atypical [1]. 46,XY gonadal dysgenesis (GD) is characterised by abnormal testicular determination. Individuals with 46,XY GD may be completely masculinised, feminised or have ambiguous genitalia. In cases of pure or complete gonadal dysgenesis (CGD), the testes are absent and bilateral streak gonads are observed, along with concomitant female internal and external genitalia. Molecular genetic analyses of individuals exhibiting 46,XY GD and CGD have played a crucial role in the identification of human testis-determining genes: *SRY* [2], *SOX9* [3], [4], *WT1* [5] and *SF1* [6] were all established as sex determining in humans on the basis of molecular lesions in individuals with 46,XY GD. However, it has been estimated that approximately 60-70% of all cases of XY GD remain unexplained at the molecular level [7].

Attempts to identify these missing human testis determining genes have recently focused on the study of familial cases of 46,XY DSD unlinked to known genes [8], [9]. In one such family, 46,XY GD was transmitted as an autosomal dominant trait with highly variable expressivity, ranging from CGD to partial GD associated with normal female genitalia, sexual ambiguity or mild hypospadias in affected males [10]. Linkage analysis in this family placed the mutated locus on the pericentric region of chromosome 5 [11]. Recently, we attributed the cause of 46,XY GD in this, and a second family [12], to mutations in the gene encoding the signal transduction molecule, MAP3K1 [13]. Two sporadic cases of 46,XY GD were also reported to harbour *MAP3K1* mutations. MAP3K1 (also known as MEK kinase 1 (MEKK1)) encodes a MAPK kinase kinase that acts as part of a phosphorelay triad to phosphorylate the MAPKs JNK, ERK and p38, with a strong preference for the JNK pathway [14], [15], [16]. The MAPK pathway acts to integrate diverse signals to regulate a variety of cellular functions such as cell cycle progression, cell adherence, motility and metabolism and thereby influence a number of developmental processes. In particular, mammalian sex determination is regulated by growth factors such as insulin-like growth factors [17], fibroblast growth factors [18], [19], [20], prostaglandins [21], [22] and platelet-derived growth factors [23]. MAP3K1 might act to regulate or integrate such signals during testis development [24].

Analysis of MAPK signalling activity in lymphoblastoid cell lines derived from individuals with sex-reversing *MAP3K1* mutations revealed enhanced phosphorylation of the MAPKs p38 and ERK after serum starvation followed by re-feeding [13]. Moreover, RHOA, a known positive regulator of MAP3K1 kinase activity, exhibited increased binding to protein complexes containing mutant MAP3K1. These data raise the possibility that, at least in the lymphoblastoid cell line context, mutant versions of *MAP3K1* behave like gain-of-function alleles, enhancing functionality of the encoded protein. This possibility is also consistent with the absence of any truncating, loss-of-function, mutations in the 46,XY GD patient cohorts examined. However, direct targets of MAP3K1 were not assayed. Moreover, crosstalk between the ERK and JNK/p38 pathways is reported to regulate apoptosis in some contexts, indicating that the distinct MAPK pathways are not insulated from each other [25]. Thus, disruption to one element of the MAPK signalling network may conceivably cause consequential activation of other components. Along with the fact that all these functional studies were performed in heterologous lymphoblastoid cell lines, these observations indicate that no definitive explanation yet exists for how these *MAP3K1* mutations disrupt human testis determination.

We have recently established a role for another MAP3K in mouse sex determination [26]. A forward genetic screen identified the boygirl (*byg*) mutation, which in homozygous embryos on the C57BL/6J background causes XY gonadal sex reversal. Positional cloning identified a point mutation in *Map3k4*, resulting in a premature stop codon and, thus, a null allele. Analysis of gonad development in XY *byg/byg* embryos revealed failure to execute the testis-determining programme due to delayed and greatly reduced levels of *Sry* expression. These data, and those implicating *MAP3K1* in human testis development, suggest a conserved role for MAPK signalling in mammalian sex determination. However, it is unclear whether sex determination in the mouse utilises MAP3K4 exclusively, or whether a role exists for MAP3K1 too. Mice lacking *Map3k1* have been described and, along with disruption to MAPK signalling, these exhibit defects in embryonic eyelid closure; but no defects in sexual development have been reported on a mixed genetic background, although no systematic study has been described [15], [16], [27], [28]. However, on the C57BL/6J background, one that is especially sensitive to disruptions to the testis determination pathway, *Map3k1*-deficient mice are non-viable due to defects in foetal erythropoiesis [29]. Thus, a careful examination of XY gonad development on this background is required to address the role of MAP3K1 in mouse testis determination and allow comparison with its role in humans. Here we show that no overt abnormalities in testis determination are observed in *Map3k1*-deficient XY embryos on C57BL/6J, although minor defects are apparent. Moreover, no additional abnormalities are observed in mice lacking *Map3k1* and a single copy of *Map3k4*, suggesting that no genetic interaction occurs between these loci during sex determination. We conclude, therefore, that MAP3K1 is not required for mouse testis determination.

## Materials and Methods

### Mouse mutants utilized and genotyping

The *Map3k1^ΔKD^* allele generates a MAP3K1-β-galactosidase fusion protein containing the first 1188 amino acids of MAP3K1, but entirely lacking the kinase domain required for its function [16]. *Map3k1^ΔKD^* mice were maintained on two distinct genetic backgrounds: C57BL/6J, by out-crossing, and a mixed C57BL/6J-C3H/HeH background by continual intercrossing. Mice and embryos were genotyped using a novel three-primer PCR assay in which mutant (216 bp) and wild-type (475 bp) products were amplified using primers: 5′-GCGATGTCGCGTCTCAGG-3′, 5′-GGCCTTTCAGCCACTCAGC-3′ and 5′-AAAGCGCCATTCGCCATT-3′. *Map3k4^tm1Flv^* ^/+^ mice [30] were maintained on C57BL/6J and genotyped as previously described [26]. Embryos were sexed by a PCR assay that simultaneously amplifies the *Ube1y1* and *Ube1x* genes as previously described [31]. All mice were bred under standard conditions of care and used under licensed approval from the UK Home Office (PPL 30/2381: Dr A Greenfield). This investigation conforms with the *Guide for the Care and Use of Laboratory Animals* published by the US National Institutes of Health (NIH Publication No. 85–23, revised 1996).

### Generation of mutant embryos and expression analyses

Noon on the day of the copulatory plug was counted as 0.5 dpc. Embryos were staged accurately based on the number of tail somites or limb and gonad morphology. Wholemount *in situ* hybridization (WMISH) analysis of embryonic tissues was performed as previously described [32], [33]. Probes for *Sox9* [34], *Oct4* and *3ßHSD* [35], *Stra8* and *Wnt4* [26] have been previously described.

Detection of the *Map3k1^ΔKD^ lacZ* reporter was performed using a protocol based on one previously described [36]. Embryos were dissected in PBS to expose the developing reproductive organs/tracts, fixed on ice (1% PFA, 0.2% glutaraldehyde, 2 mM MgCl_2_, 5 mM EGTA, 0.02% NP-40 in PBS) and then washed in PBS/0.02% NP-40. Staining was carried out in the dark, at room temperature for 16 hrs or until blue colour fully developed in X-gal stain (PBS containing 5 mM K_3_Fe(CN)_6_, 5 mM K_4_Fe(CN)_6_, 2 mM MgCl_2_, 0.01% deoxycholate, 0.02% NP-40, 1 mg/ml X-Gal). Samples were post-fixed in 4% PFA/PBS.

### Immunohistochemistry and Western blotting

A commercially available antibody was used to detect AMH (MIS (C-20):sc-6886, *Santa Cruz*). Immunostaining was performed on wax sections and imaged using a Leica TCS SP5 confocal as previously described [26], [37]. Antibodies against p38 (#9212, *Cell Signaling*), phospho-p38 (pp38) (#4631, *Cell Signaling*) and ß-actin (A 2066, *Sigma*) were used for Western blotting. Gonads from control and mutant 12.5 dpc embryos were lysed in Tris-buffered saline (pH8) containing 1% NP40 and a cocktail of protease and phosphatase inhibitors (cOmplete mini 04 693 124 001 Roche, PhosSTOP 04 906 837 001 Roche and sodium orthovanadate P078S New England BioLabs). 2 µg from each protein sample was loaded on NuPAGE 4–12% Bis/Tris gel (Invitrogen), blotted onto nitrocellulose membrane (Invitrogen) and blocked in Tris-buffered saline containing 0.1% tween-20 and 5% dry skimmed milk. Antibodies were diluted in blocking solution at 1∶1000 (anti-p38 and -pp38), 1∶2000 (anti-actin) and 1∶3000 (anti-rabbit-HRP). Incubation with primary antibodies proceeded overnight at 4°C and with secondary antibody for 1 hour at room temperature. ECL or ECL Plus systems (GE Healthcare) were used for detection.

## Results

### *Map3k1* is expressed in male and female gonads at the sex determining stage of gonadogenesis

**Figure 1:**
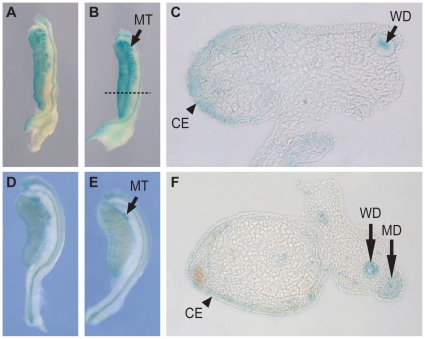
*Map3k1* is expressed most strongly in the developing gonadal coelomic region as revealed by X-gal staining of the *Map3k1^ΔKD^* reporter. (A–C) X-gal staining in XX (A) and XY (B) gonads at 11.5 dpc. (C) Transverse section of gonad in (B) at level indicated by dashed line, revealing expression in the coelomic epithelium and subepithelial mesenchyme. (D–F) Staining of 13.5 dpc XX (D) and XY (E) gonads. (F) Transverse section of 13.5 dpc testis. CE, coelomic epithelium; WD, Wolffian duct; MD, Müllerian duct.

We first analysed expression of *Map3k1* in the developing gonads by exploiting a *lacZ* reporter engineered into the targeted *Map3k1^ΔKD^* allele [16]. Examination of ß-galactosidase activity in the gonads of *Map3k1^ΔKD^*/+ embryos revealed weak *lacZ* reporter activity in both the XX and XY gonads at 11.0 dpc in the gonad, Wolffian duct and mesonephric tubules, but no staining was detected prior to this at 10.5dpc (data not shown). At 11.5 dpc, the sex-determining stage of gonad development, prominent expression was observed throughout the gonad with the highest levels in the coelomic region at the periphery of the gonad in both XY and XX gonads (Figure. 1A–C). By 13.5 dpc signal was detected in the coelomic region of the testis and in the developing reproductive tracts and mesonephric tubules (Figure. 1D–F). We have previously described WMISH analysis with a *Map3k1* probe and the profile of expression obtained was identical with that of the *lacZ* reporter at the sex determining stage (11.5 dpc). However, in contrast to the reporter profile, *in situ* analysis revealed prominent expression in the testis cords at 13.5 dpc, in a pattern indicative of Sertoli cell expression [13] . No sexual dimorphism was observed using *in situ* hybridisation or reporter profiling prior to sexual differentiation of the gonads and at no stage was the level of expression noticeably higher in XY gonads.

### *Map3k1*-deficient mice are viable and fertile on a mixed genetic background

We first analysed homozygotes for the *Map3k1^ΔKD^* allele on a mixed genetic background (see Materials and Methods). All homozygotes exhibited the eyes open at birth (EOB) phenotype, as previously described [27], [28]. They were otherwise healthy in appearance and behaviour. We examined fertility in homozygous males (n = 5) by performing test crosses to wild-type female mice. All male homozygotes tested generated a copulatory plug and yielded litters of average size 6.8 (n = 20 litters), examined by performing openings at mid-gestation. Post-mortem analysis of the reproductive organs in homozygous males revealed the presence of motile sperm in the epididymis (n = 7) and no reduction in testis size (mean = 0.11 g STD = 0.02 n = 12) when compared to age-matched controls (mean = 0.09 g STD = 0.01 n = 7). Histological examination of sections revealed numerous seminiferous tubules containing germ cells. The vas deferens, seminal vesicles and accessory glands also appeared normal (data not shown). From these data we conclude that MAP3K1 is not required for testis determination or testis functioning on a mixed genetic background.

### Analysis of *Map3k1^ΔKD/ΔKD^* homozygotes on C57BL/6J reveals normal testis determination but increased embryonic gonadal length

**Figure 2:**
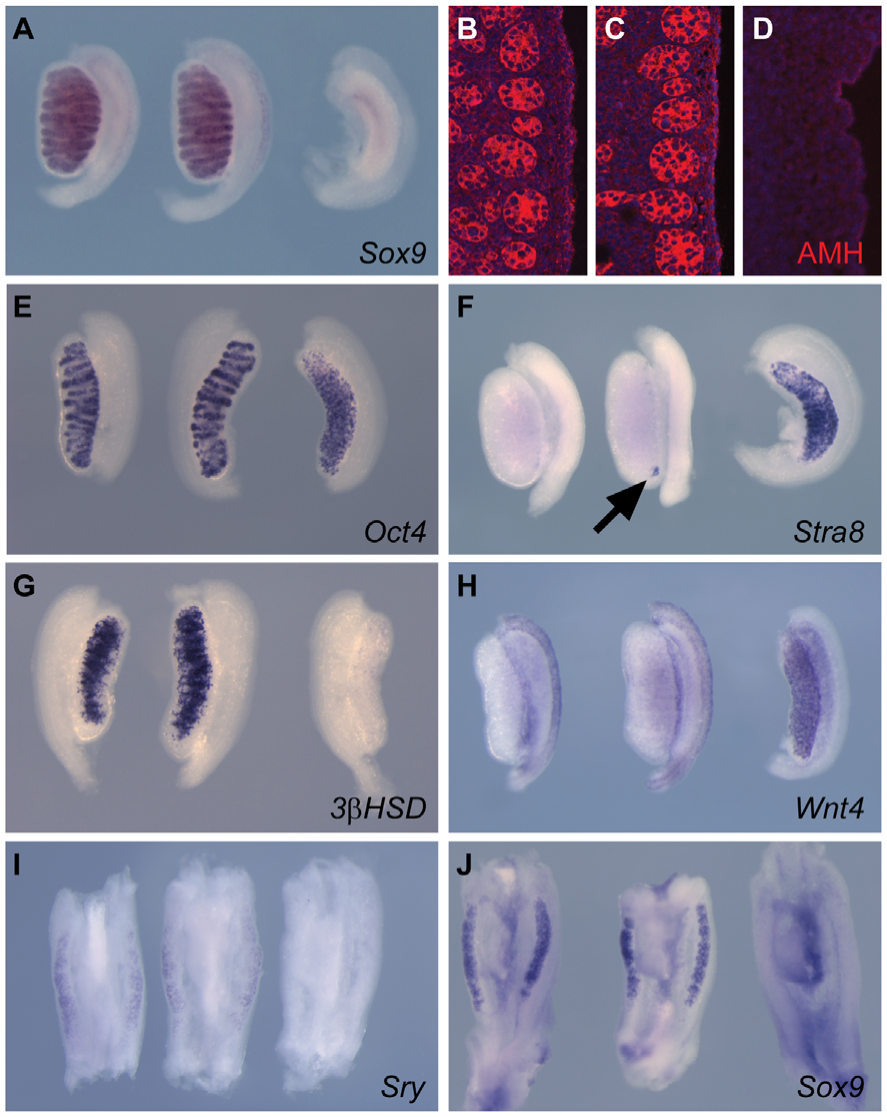
Marker analysis of *Map3k1^ΔKD/ΔKD^* XY embryonic gonads reveals minor defects in male development. WMISH and immunofluorescence were performed at 14.5 dpc (A–D, F & H), 13.5 dpc (E & G) and 11.5 dpc (I–J) using the indicated markers of sexual development. Respective genotypes are arranged in the following left-to-right order: XY *Map3k1^+/+^;* XY *Map3k1^ΔKD/ΔKD^;* XX *Map3k1^+/+^*. (A–D) *Sox9* and AMH are markers of the Sertoli cell lineage and highlight the testis cords. (E) *Oct4* marks germ cells in both sexes. Note increased length of the *Map3k1^ΔKD/KD^* gonad (centre) and the disorganised cord structure at the caudal pole. (F) *Stra8* marks meiotic germ cells, normally only present in embryonic ovaries. Arrow shows positive cells in XY *Map3k1^ΔKD/ΔKD^* testis. (G) *3ß–HSD* is a Leydig cell marker. (H) *Wnt4* is a marker of ovarian somatic cells at this stage. (I) *Sry* (a marker of Sertoli cell precursors) expression appears unaltered at the sex-determining stage. (J) Expression of the testis-determining gene *Sox9,* at 11.5dpc, appears normal in XY *Map3k1^ΔKD/ΔKD^* gonads.

C57BL/6J (B6) is a genetic background known to be sensitised to disruptions to testis development [26], [38], and therefore we examined testis development in *Map3k1^ΔKD/ΔKD^* homozygotes after backcrossing the *Map3k1^ΔKD^* allele to this inbred strain for between two to four generations. As previously reported, *Map3k1*-deficient mice are mostly non-viable on this background [29]. After intercrossing heterozygous animals, a significant loss of homozygotes was observed at weaning. Intercrossing backcross-three heterozygous animals resulted in just 4.3% (6 out of 140) homozygous animals. Openings at 14.5 dpc revealed homozygous embryos with a pale appearance due to anaemia and exhibiting necrosis. Given the frequency of dead and dying homozygous embryos at 14.5 dpc (45%), we analysed mutant embryos at 14.5 dpc (if still alive) and 13.5 dpc. Anaemic homozygous embryos were still detected at 13.5 dpc, but in reduced numbers (the frequency of dead embryos was 10%) and they showed no signs of tissue necrosis. Chromosomal typing (XY versus XX) revealed no discordance between chromosomal and gonadal sex in these embryos. Examination of the embryonic testes at these stages (n = 26) with markers of distinct cell lineages, including Sertoli cells (*Sox9*, AMH), germ cells (*Oct4*, *Stra8*), Leydig cells (*3-ßHSD*) and ovarian somatic cells (*Wnt4*) revealed no obvious failure of the testicular programme of differentiation (Figure. 2A–H), although, occasionally, small clusters of *Stra8*-positive cells were observed in homozygous testes at the caudal pole (see, for example, Figure. 2F). *Stra8* marks meiotic germ cells, normally absent from the testis at this stage. Curiously, homozygous mutant testes at these stages consistently appeared longer than controls (see, for example, Figure. 2E–H). Careful measurement of gonadal length in stage-matched embryos revealed that mutant testes at 13.5 dpc were on average 16% longer than wild-type controls (n = 8 stage-matched pairs, STD  = 4.73%). No correlative lengthening of *Map3k1^ΔKD/ΔKD^* embryos was observed.

In order to examine whether gene expression profiles in *Map3k1*-deficient gonads were normal at 11.5 dpc, the sex-determining stage of gonadogenesis, we analysed the expression of *Sry* (Figure. 2I), *Sox9* (Figure. 2J) and *Wnt4* (data not shown); no overt differences were observed between mutants and controls (n = 16 embryonic testes). Due to the reported increased phosphorylation of p38 in lymphoblastoid cell lines derived from patients harbouring mutations of human *MAP3K1* [13], we also examined levels of phospho-p38 (pp38) in control and homozygous mutant gonads at 12.5 dpc (Figure. S1). We observed no significant difference in levels of pp38 between mutant and control samples.

### Incompletely penetrant imperforate vagina phenotype in adult XX *Map3k1^ΔKD/ΔKD^* homozygotes

**Figure 3:**
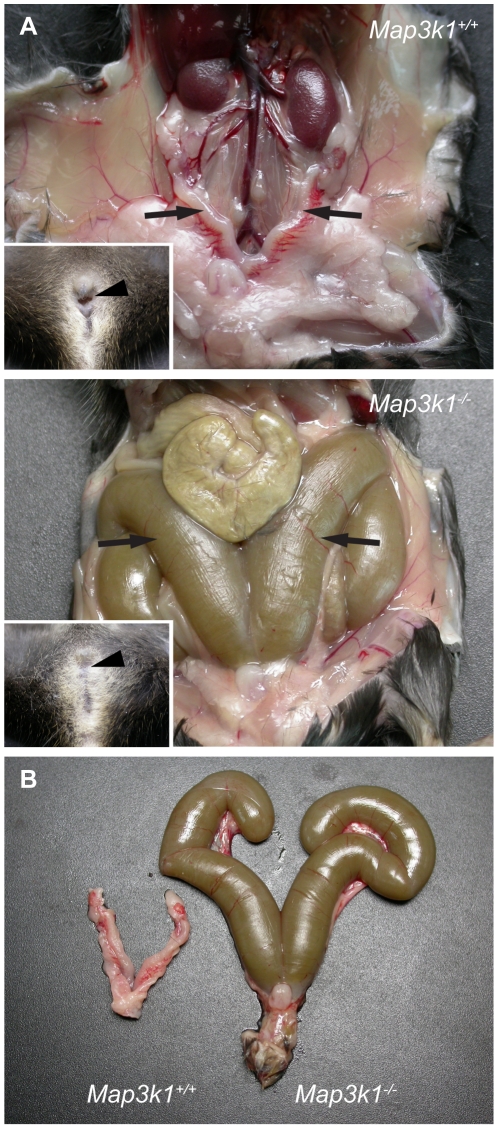
XX *Map3k1* *^ΔKD/ΔKD^* females on C57BL/6J display imperforate vagina. (A) Comparison of XX *Map3k1^+/+^* (upper panel) and XX *Map3k1^ΔKD/ΔKD^* (lower panel) reproductive tracts reveals grossly distended and swollen uterine horns (arrows) in the mutant, compared to wild-type (arrows). Mutants lack a vaginal opening, which is overt in wild-type individuals (compare arrowheads in inset images). (B) Dissected reproductive tracts allow the increased size of the mutant uterine horns to be observed more clearly.

We also examined the rare, viable homozygotes that survived to adulthood. Again, there was no discordance between chromosomal and phenotypic sex in these animals. Two homozygous mutant males were shown to be fertile and histological examination of testis sections revealed no obvious abnormalities. Interestingly, four homozygous mutant females (out of a total of ten) exhibited imperforate vagina (Figure. 3). At necropsy, this was shown to be associated with grossly distended uterine horns. Imperforate vagina was not observed on the mixed genetic background studied here. Two females not exhibiting imperforate vagina were also fertility tested, and between them yielded five litters, with an average of six pups per litter. Recently, it was reported that mice lacking BCL-2 modifying factor (BMF), a member of the BH3-only group of proapoptotic proteins, exhibit defects in uterovaginal development, including an imperforate vagina [39]. BMF is a target for phosphorylation by JNK, thus MAP3K1 may act upstream of JNK signaling during uterovaginal tissue morphogenesis.

### Investigation of potential genetic interaction between *Map3k1* and *Map3k4* in testis determination (on C57BL/6J)

**Figure 4:**
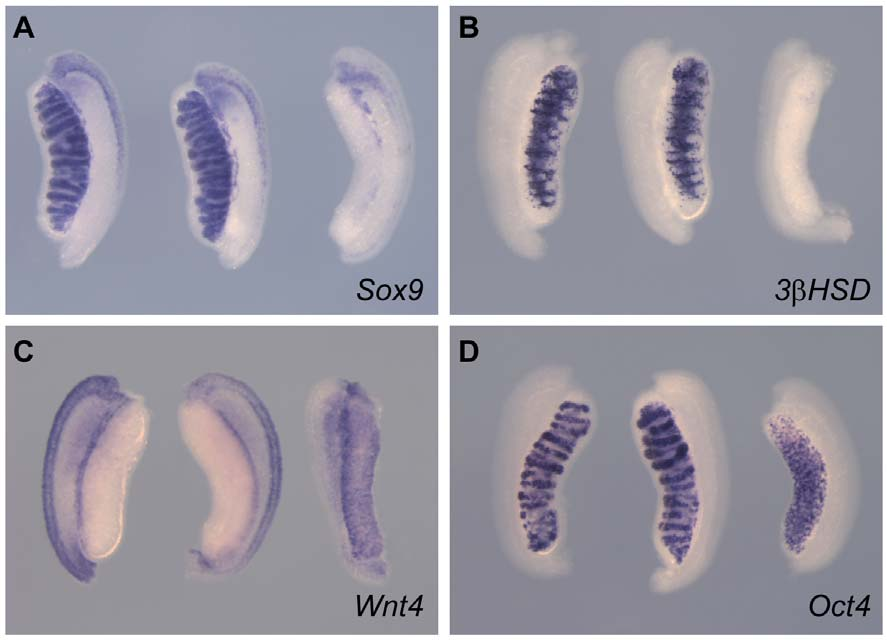
*Map3k1* does not function redundantly with *Map3k4* during sex determination. WMISH was performed at 13.5 dpc using the following probes: (A) *Sox9*, (B) *3ß-HSD*, (C) *Wnt4,* (D) *Oct4*. Respective genotypes are arranged in the following left-to-right order: XY *Map3k1^ΔKD/ΔKD^, Map3k4^+/+^;* XY *Map3k1^ΔKD/ΔKD^*, *Map3k4^tm1Flv/+^*; XX *Map3k1^ΔKD/ΔKD^, Map3k4^+/+^.* Following marker analysis, the expression patterns observed using XY compound mutant gonads were indistinguishable from those of controls.

The observation that absence of MAP3K1 is compatible with the formation of functional testes on a mixed genetic background and testis determination and differentiation on B6 suggests the possibility of functional redundancy, at least during sexual development. We performed a genetic test to determine whether MAP3K4 might be able to compensate for the absence of MAP3K1 during sex determination by generating mice and embryos lacking both copies of *Map3k1* and a single copy of *Map3k4,* using a targeted *Map3k4* null allele [30]. We have previously shown that XY mice lacking a single copy of *Map3k4* (*Map3k4^tm1Flv/+^*) can develop ovotestes or, occasionally, ovaries when on a highly sensitised genetic background (B6-Y^AKR^) [26], suggesting that *Map3k4* is a dosage-sensitive locus in the appropriate genetic context. We were unable to generate viable adults of the genotype *Map3k1^ΔKD/ΔKD^*, *Map3k4^tm1Flv/+^* mice on B6, presumably due to the lethality associated with homozygosity for *Map3k1^ΔKD^*. However, XY embryos of this genotype examined at 13.5 dpc developed as males with a grossly normal testicular morphology, although still exhibiting the gonadal lengthening associated with homozygosity for *Map3k1^ΔKD^* (Figure. 4). Marker analyses of these testes (n = 8) revealed no abnormalities when compared to controls (here, *Map3k1^ΔKD/ΔKD^* embryos). Moreover, XX *Map3k1^ΔKD/ΔKD^* homozygous gonads exhibited a normal ovarian morphology and normal expression of the markers *Sox9*, *3ß-HSD*, *Wnt4* and *Oct4* (Figure. 4), consistent with the fertility of adult females on this background, described above. We conclude that there is no overt genetic interaction between *Map3k1* and *Map3k4* during testis determination in the mouse.

## Discussion

Here, we report that absence of MAP3K1 does not disrupt mouse testis determination on either a mixed genetic background or on the sensitised B6 background. Whilst it was not possible to assess testis function on B6 due to non-viability, marker analysis of XY embryonic gonads on this background indicates that primary sex determination occurs as normal, with all major testicular lineages present and no evidence of inappropriate, widespread ovarian marker expression. Expression of the *Map3k1^ΔKD^* reporter is consistent with a role in sex determination and male and female sexual development more broadly. Expression is first observed at 11.0 dpc, and is prominent at 11.5 dpc, the sex-determining stage of gonad development. Highest levels of expression at this stage are detected in the coelomic epithelium and sub-epithelial mesenchymal cells. The significance of this profile is unclear, although the coelomic epithelium is the source of at least a subset of Sertoli cells [40] and male-enhanced cell proliferation in this region is one of the first consequences of SRY expression [41]. Reporter signal is diminished by 13.5 dpc and is restricted to the coelomic epithelium. This observation is in contrast to *Map3k1 in situ* hybridisation analysis, which reveals expression in the ovary and testis cords at 13.5 dpc, consistent with expression in pre-Sertoli cells [13]. This discrepancy might be explained by the loss of regulatory elements in the targeted *Map3k1^ΔKD^* allele. Other aspects of the *in situ* and reporter expression profiles, including expression in the developing reproductive tracts, are in agreement, suggesting that the probe utilised for *in situ* hybridisation is of good quality.

One abnormality that was observed in *Map3k1^ΔKD^* homozygous embryos was an increased length of mutant gonads at 13.5 dpc. The cellular basis for this phenotype is unclear, however, it might explain the presence of small clusters of *Stra8*-positive cells at the caudal pole of some mutant gonads. The program of testis development is thought to radiate from initial SRY-dependent events in the centre of the gonadal primordium. This centre-to-pole masculinisation is thought to be driven by the secreted molecule, FGF9 [42]. Any delay in the receipt of this signal by the gonadal poles can result in the formation of ovarian tissue in this region, as characterised by ovotestis development, due to antagonism of the testis-determining pathway by ovarian-determining genes [43]. The increase in the length of the mutant gonads reported here might result in occasional delay in the masculinising signal reaching the poles and subsequent entry of XY primordial germ cells into meiosis. Notably, successful testis determination in *Map3k1^ΔKD^* homozygotes is not overtly disrupted by the further removal of a single copy of *Map3k4,* a known dosage-sensitive sex-determining gene in the mouse that also functions in the MAPK signalling pathway to activate JNK and p38 [44] [26], [45]. Thus, we conclude that MAP3K1 is not required for testis determination in the mouse, potentially highlighting a difference between mice and humans with respect to MAP3K1 and its role in sex determination. What might account for this apparent discrepancy?

Firstly, the established involvement of MAP3K1 in human sex determination, and MAP3K4 in mouse sex determination, strongly suggest a conserved role for MAPK signalling in mammalian sex determination, potentially in the regulation of the activity of target proteins e.g. transcription factors. However, it is possible that whilst a conserved signal transduction pathway exists, these pathways are divergent at the MAP3K level of functionality: humans employ MAP3K1 (and presumably other interacting molecules) to regulate this pathway, but there are no reports of mutations in *MAP3K4* causing human DSDs. In contrast, mice employ MAP3K4 in testis determination and not, as we demonstrate here, MAP3K1. If this hypothesis of a conserved role for MAPK signalling is correct, there must be convergence of the pathway in mice and humans at the level of MAP2K, MAPK or target protein functionality and this can be tested by the identification of sex reversing MAP2K or MAPK mutations in both mice and humans, or mutations in MAPK target genes. A systematic genetic analysis is complicated by the potential number of genes involved at this level of the MAPK pathway. MAP3K4 can activate the MAPKs p38 and JNK via the phosphorylation of the MAP2Ks MKK3/MKK6 and MKK4/MKK7, respectively [44], [45]. MAP3K1 can phosphorylate MKK4 to activate JNK and MKK1 to activate ERK1/2 [15], [46]. Thus, several MAP2Ks may play a role. Moreover, there are three mammalian genes encoding JNK, and four encoding p38 isoforms [47].

A second way of explaining the apparent discrepancy between the roles of *MAP3K* genes in mice and humans involves the nature of the human mutations themselves. Functional studies in mice, such as that described here, invariably involve loss-of-function alleles generated by gene targeting. However, the spectrum of alleles contributing to human genetic disease is much greater, and this may underlie the difference between humans and mice harbouring *MAP3K1* mutations. It is notable that none of the *MAP3K1* mutations reported to disrupt human testis development are truncating i.e. none encode early termination codons in the MAP3K1 polypeptide [13]. This suggests that these mutations do not generate null alleles, possibly reflecting a loss of viability in humans exhibiting widespread absence of MAP3K1 function, as in mice on the B6 background. Assays in lymphoblastoid cell lines revealed increased phosphorylation of the MAPKs, p38 and ERK. How increased activity of MAPKs could disrupt testis determination, in the absence of known MAPK target proteins that function in sex determination, is, however, unclear. Disruption to one or more protein interactions of MAP3K1, which are numerous [48], may contribute to the sex-reversal phenotype. It is also worth noting that putative MAP3K1 gain-of-function during XY female development might be interpreted as implying a positive role for MAP3K1 in ovary development itself. However, on C57BL/6J, XX *Map3k1^ΔKD/ΔKD^* homozygous adult females (n = 2) were fertile and homozygous XX embryos exhibited no overt abnormalities of ovary development. Thus, there appears to be no requirement for MAP3K1 in ovary determination in mice.

Finally, the possibility of unconventional roles for MAP3K1 in sex determination cannot be excluded. MAP3K1 differs from MAP3K2, MAP3K3 and MAP3K4 in exhibiting lower levels of conservation in its kinase domain and in being a strong stimulator of apoptosis [49]. MAP3K1 is a 196-kDa protein that encodes a protease cleavage sequence for caspase-3-like proteases and UV irradiation and DNA-damaging chemicals activate MAP3K1 kinase function and induce its proteolytic cleavage [50]. These data suggest that MAP3K1 is an integral component of the apoptotic response. We cannot exclude disruption to this or other unconventional functions of MAP3K1 as causes of human sex reversal. Further careful experimentation will be required to appropriately address these complex issues.

## Supporting Information

Figure. S1 **Levels of phosphorylated p38 (pp38) are not altered in 12.5 dpc gonads of** ***Map3k1^ΔKD/ΔKD^*** **embryos.** (A) Western blot analysis of protein samples from sub-dissected 12.5 dpc embryonic gonads using the antibodies indicated. ß-actin detection was used as a loading control. pp38 and p38 levels appear very similar in homozygous mutants (homo) and controls. (B) Graphical representation of the average normalised levels of pp38 and p38 (derived from two independent pairs of samples (four gonads)). Two-tailed t-tests confirm that the levels are not significantly different between samples (pp38, p = 0.393; p38, p = 0.686).
(TIFF)
