# Supplementary figures and images for: Minor Abnormalities of Testis Development in Mice Lacking the Gene Encoding the MAPK Signalling Component, MAP3K1
Source: PLoS One. 2011 May 3;6(5):e19572. doi: 10.1371/journal.pone.0019572 (PMC3086927; doi:10.1371/journal.pone.0019572)

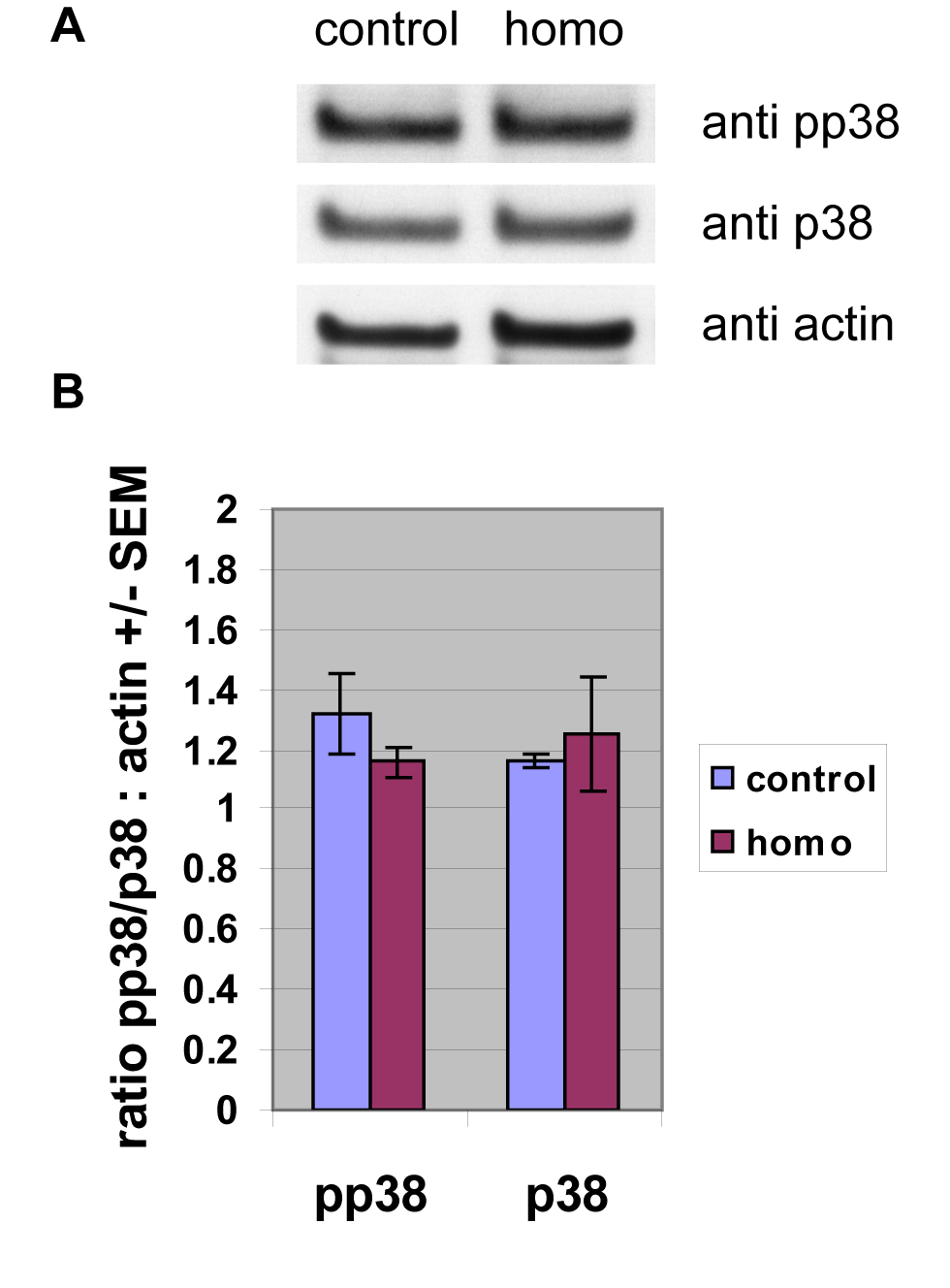

Supplement: Figure. S1 — Levels of phosphorylated p38 (pp38) are not altered in 12.5 dpc gonads of Map3k1ΔKD/ΔKD embryos. (A) Western blot analysis of protein samples from sub-dissected 12.5 dpc embryonic gonads using the antibodies indicated. ß-actin detection was used as a loading control. pp38 and p38 levels appear very similar in homozygous mutants (homo) and controls. (B) Graphical representation of the average normalised levels of pp38 and p38 (derived from two independent pairs of samples (four gonads)). Two-tailed t-tests confirm that the levels are not significantly different between samples (pp38, p = 0.393; p38, p = 0.686). (TIFF) [file pone.0019572.s001.tif]
